# Supplementary material for: PAK6 rescues pathogenic LRRK2-mediated ciliogenesis and centrosomal cohesion defects in a mutation-specific manner
Source: Cell Death Dis. 2024 Oct 17;15(10):752. doi: 10.1038/s41419-024-07124-4 (PMC11487180; doi:10.1038/s41419-024-07124-4)
Supplement: Supplementary file 1 — Supplementary material file [file 41419_2024_7124_MOESM1_ESM.pdf]

PAK6 rescues pathogenic LRRK2-mediated ciliogenesis and centrosomal cohesion defects in a mutation-specific manner

Lucia Iannotta[1,10], Rachel Fasiczka[2], Giulia Favetta[1], Yibo Zhao[3], Elena Giusto[4], Elena Dall'Ara[1,5], Jianning Wei[6], Franz Y. Ho[5], Claudia Ciriani[1], Susanna Cogo[1, 11], Isabella Tessari[1], Ciro Iaccarino[7], Maxime Liberelle[8], Luigi Bubacco[1,9], Jean-Marc Taymans[8], Claudia Manzoni[3], Arjan Kortholt[5], Laura Civiero[1,4], Sabine Hilfiker[2,CA], Michael L. Lu[6,CA] and Elisa Greggio[1,9,CA]

[1] Department of Biology, University of Padova, Italy

[2] Department of Anesthesiology, Rutgers New Jersey Medical School, Newark, USA

[3] University College London, School of Pharmacy, London, United Kingdom.

[4] IRCCS San Camillo Hospital, Venice, Italy

[5] Department of Cell Biochemistry, University of Groningen, Groningen, Netherlands

[6] Department of Biomedical Science, Florida Atlantic University, Boca Raton, FL, USA

[7] Department of Biomedical Sciences, University of Sassari, Sassari, Italy

[8] Université de Lille, INSERM, CHU Lille, LilNCog - Lille Neuroscience & Cognition, Lille, France

[9] Centro Studi per la Neurodegenerazione (CESNE), University of Padova, Italy

[10] Current address: National Research Council, c/o Humanitas Research Hospital, Institute of Neuroscience, Rozzano, Italy

[11] Current address: School of Biological Sciences, University of Reading, Reading, United Kingdom

[CA] Correspondence to: Elisa Greggio (elisa.greggio@unipd.it); Michael Lu (MLU3@health.fau.edu); Sabine Hilfiker (sn656@njms.rutgers.edu)

Figure S1. Knockdown of PAK6 in HEK293T cells. Western blot analysis of stable HE293T cells downregulated with shRNA against human PAK6 or scramble control.

Figure S2. MLi-2 treatment rescues G2019S LRRK2-associated ciliogenesis defects in primary astrocytes

Quantification of the percentage of ciliated G2019S LRRK2 KI primary astrocytes treated with DMSO (n=62), MLi-2 10 nM (n=62) or MLi-2 200 nM (n=62). One-way ANOVA with Tukey's post-hoc test, ***$P < 0.001$; ****$P < 0.0001$.

Figure S3. No effect of PAK6 and PAK6-KM on centrosome cohesion in control or wt-LRRK2-expressing cells.

(a) Quantification of the percentage of non-transfected cells (ctrl), or cells expressing PAK6 or PAK6-KM and -/+ MLi2 treatment as indicated where duplicated centrosomes are > 2.5 µm apart (split centrosomes). Bars represent mean ± s.e.m. (n=3 experiments).

(b) Quantification of the percentage of non-transfected cells (ctrl), or cells co-transfected with wt-LRRK2 and pCMV (EV), PAK6 or PAK6-KM and -/+ MLi2 treatment as indicated which display duplicated split centrosomes. Bars represent mean ± s.e.m. (n=3 experiments).

Figure S4. PAK6 partially displaces centrosomal pRab10 in cells expressing G2019S LRRK2 but not R1441C LRRK2

(a) Example of A549 cells co-transfected with GFP (pseudo-colored blue) and flag-tagged G2019S LRRK2 and treated with DMSO or MLi-2 (200 nM, 2 h) before staining with γ-tubulin (pseudo-colored green) and pRab10 (far-red).

(b) Example of A549 cells co-transfected with tagged G2019S LRRK2 and tagged PAK6 or PAK6-KM and stained with antibodies to detect tagged PAK6 (blue), γ-tubulin (green) and pRab10 (far-red).

(c) Same as (a), but cells co-transfected with GFP and tagged R1441C LRRK2.

(d) Same as (b), but cells co-transfected with tagged PAK6 or PAK6-KM and tagged R1441C LRRK2. Arrows point to centrosomes in transfected cells. Scale bars, 10 µm. Co-localization of pRab10 and γ-tubulin as quantified from 50-60 transfected cells per condition: G2019S+GFP: 68%, G2019S+GFP+MLi2: 0%; G2019S+PAK6: 55%; G2019S+PAK6-KM: 54%; R1441C+GFP: 78%; R1441C+GFP+MLi2: 0%; R1441C+PAK6: 79%; R1441C+PAK6-KM: 73%.

(e) Proposed model of PAK6-mediated protection toward LRRK2 G2019S but not R1441C LRRK2 based on this current study and (35).

Figure S1

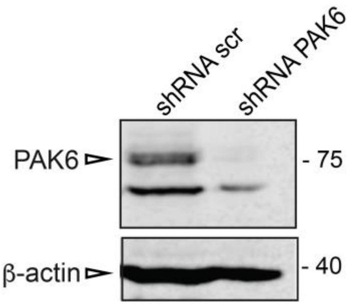

Figure S2

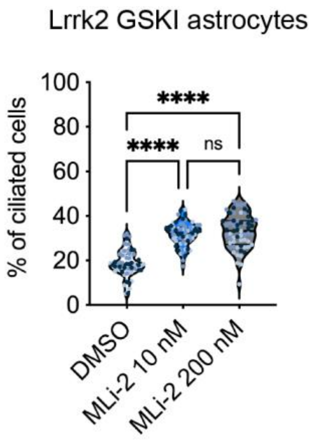
Lrrk2 GSKI astrocytes
% of ciliated cells
100
80
60
40
20
0
****
****
ns
DMSO
MLi-2 10 nM
MLi-2 200 nM

Figure S3

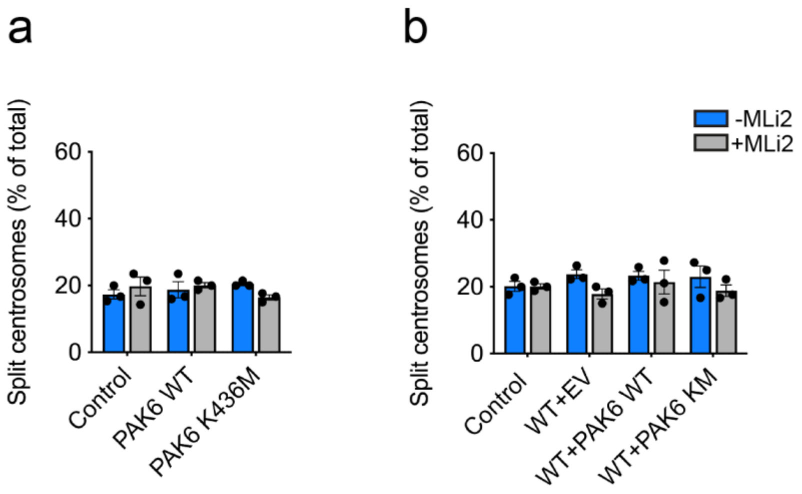
a
b
Split centrosomes (% of total)
60
40
20
0
Control
PAK6 WT
PAK6 K436M
-MLi2
+MLi2
WT+EV
WT+PAK6 WT
WT+PAK6 KM

Figure S4

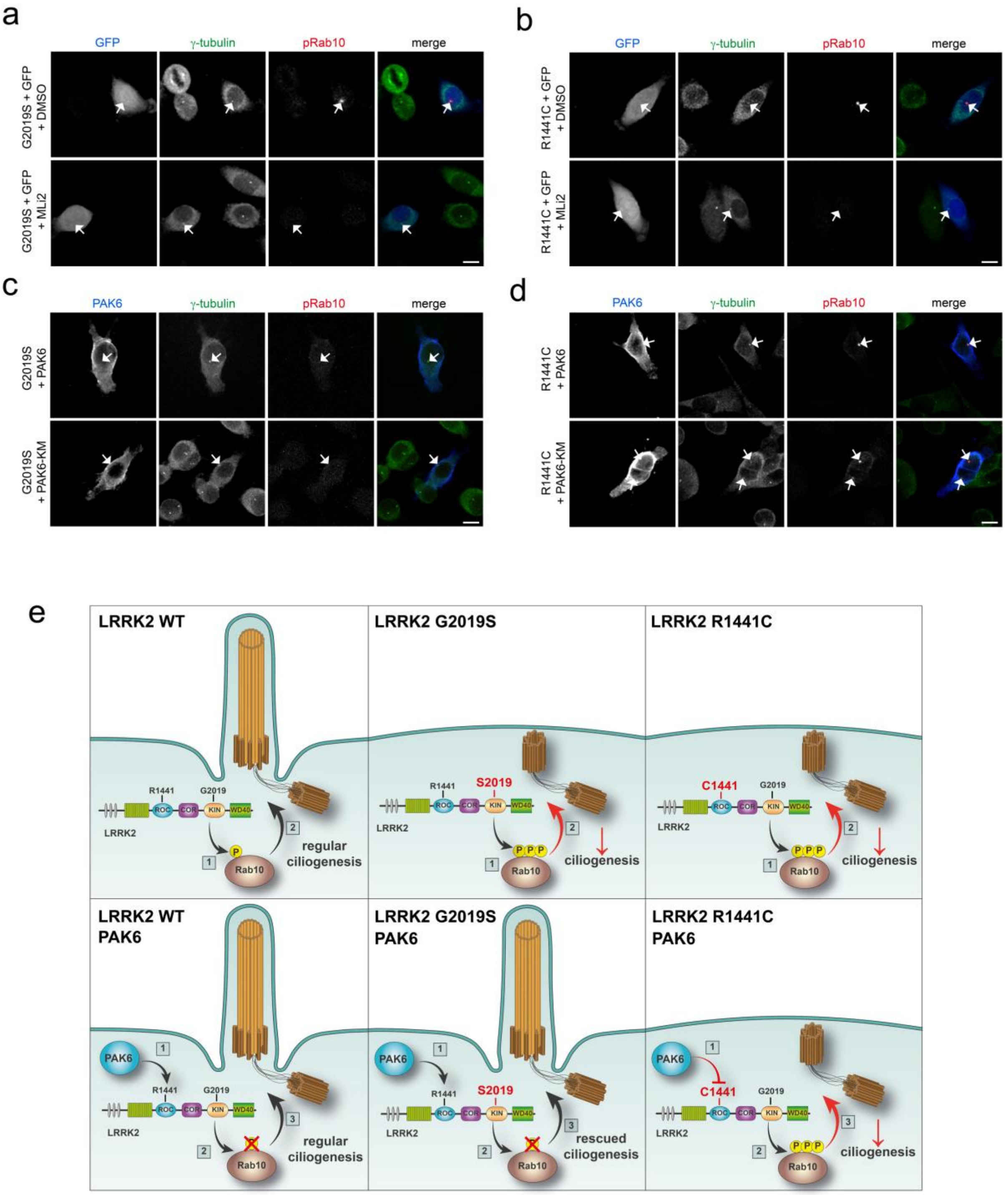

a
GFP
γ-tubulin
pRab10
merge
G2019S + GFP + DMSO
G2019S + GFP + MLi2
b
GFP
γ-tubulin
pRab10
merge
R1441C + GFP + DMSO
R1441C + GFP + MLi2
c
PAK6
γ-tubulin
pRab10
merge
G2019S + PAK6
G2019S + PAK6-KM
d
PAK6
γ-tubulin
pRab10
merge
R1441C + PAK6
R1441C + PAK6-KM
e
LRRK2 WT
LRRK2 G2019S
LRRK2 R1441C
R1441
G2019
S2019
C1441
ROC
COR
KIN
WD40
LRRK2
Rab10
P
regular ciliogenesis
ciliogenesis
LRRK2 WT PAK6
LRRK2 G2019S PAK6
LRRK2 R1441C PAK6
PAK6
rescued ciliogenesis
1
2
3
