# Supplementary figures and images for: PAK6 rescues pathogenic LRRK2-mediated ciliogenesis and centrosomal cohesion defects in a mutation-specific manner
Source: Cell Death Dis. 2024 Oct 17;15(10):752. doi: 10.1038/s41419-024-07124-4 (PMC11487180; doi:10.1038/s41419-024-07124-4)

Original Western blots


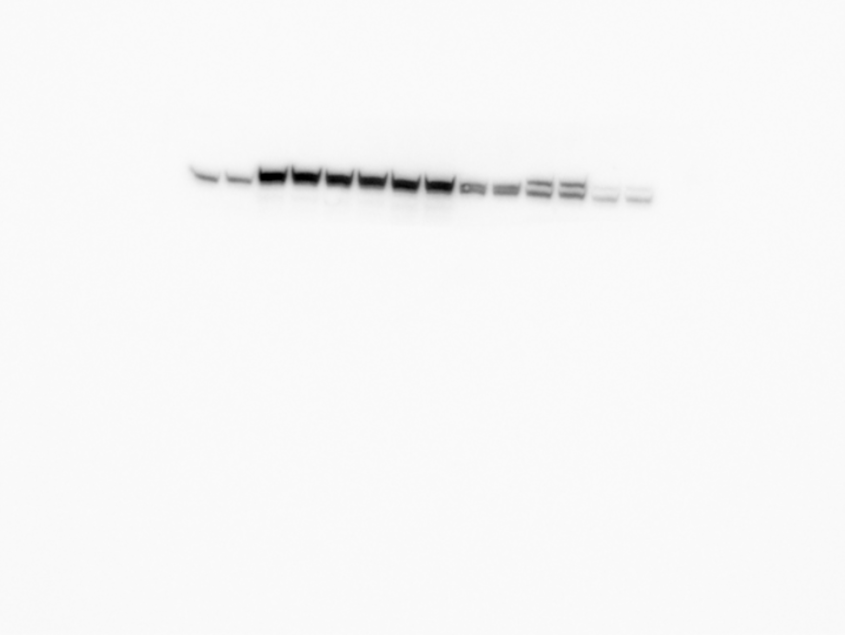


IB: pPAK 4/5/6


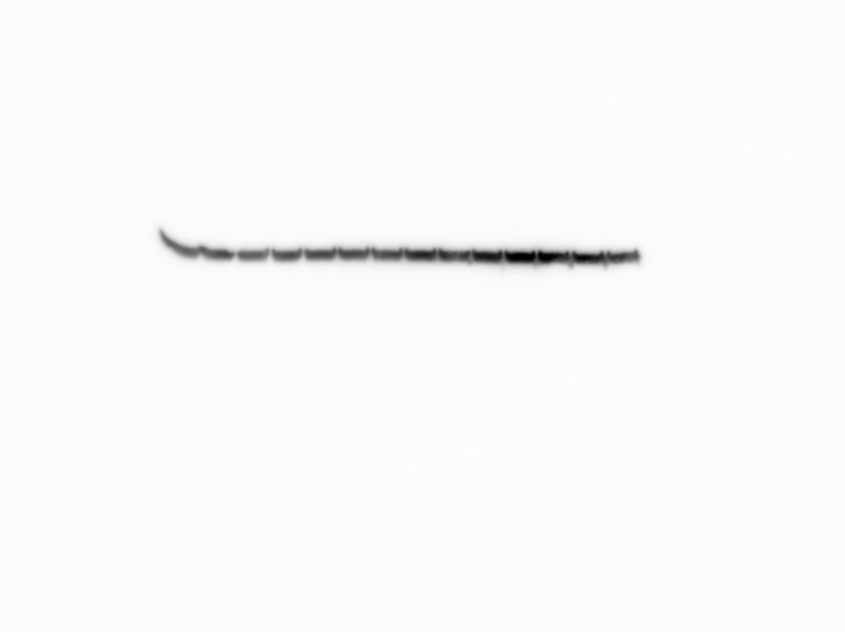

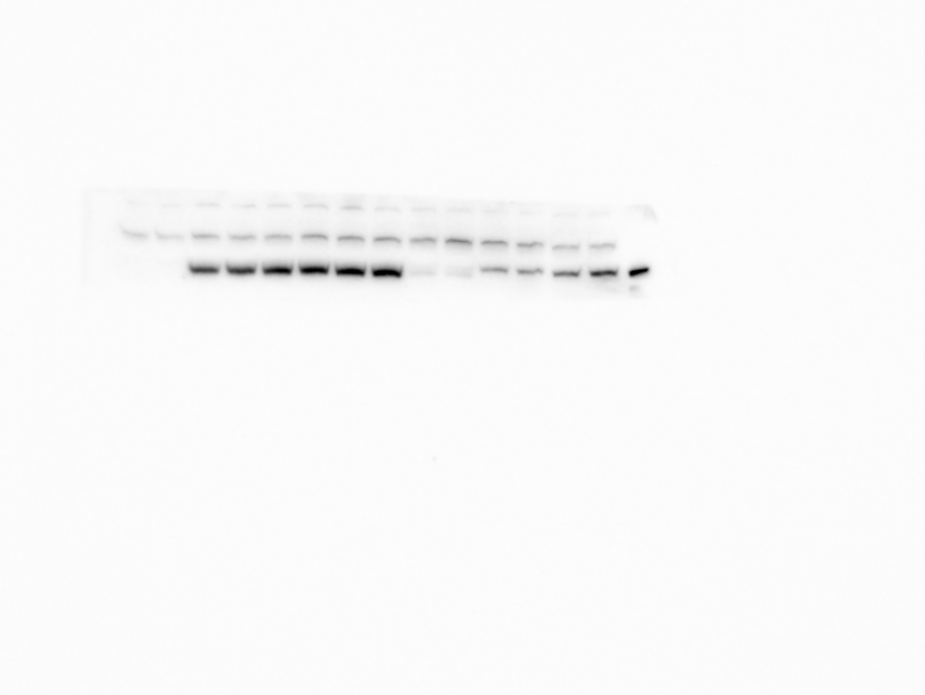


IB: PAK6

IB: b-actin


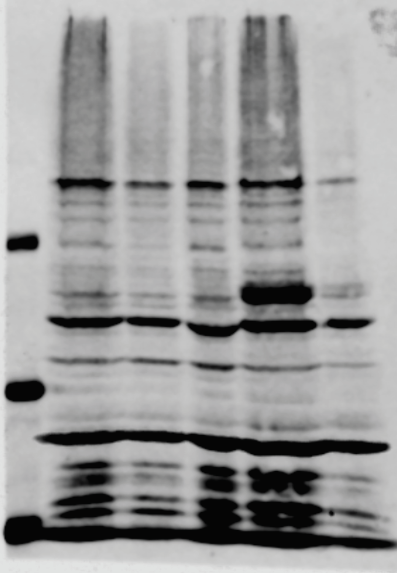


IB: b-actin

IB: PAK6

Supplement: Supplementary file 5 — Original Western blots [file 41419_2024_7124_MOESM5_ESM.docx]
